# Architectural groups of a subtelomeric gene family evolve along distinct paths in *Candida albicans*

**DOI:** 10.1093/g3journal/jkac283

**Published:** 2022-10-21

**Authors:** Matthew J Dunn, Shahed U A Shazib, Emily Simonton, Jason C Slot, Matthew Z Anderson

**Affiliations:** Department of Microbiology, The Ohio State University, Columbus, OH 43210, USA; Department of Microbiology, The Ohio State University, Columbus, OH 43210, USA; Department of Microbiology, The Ohio State University, Columbus, OH 43210, USA; Department of Plant Pathology, The Ohio State University, Columbus, OH 43210, USA; Department of Microbiology, The Ohio State University, Columbus, OH 43210, USA; Department of Microbial Infection and Immunity, The Ohio State University, Columbus, OH 43210, USA

**Keywords:** gene families, gene diversification, subtelomeres, Mediator

## Abstract

Subtelomeres are dynamic genomic regions shaped by elevated rates of recombination, mutation, and gene birth/death. These processes contribute to formation of lineage-specific gene family expansions that commonly occupy subtelomeres across eukaryotes. Investigating the evolution of subtelomeric gene families is complicated by the presence of repetitive DNA and high sequence similarity among gene family members that prevents accurate assembly from whole genome sequences. Here, we investigated the evolution of the telomere-associated (*TLO*) gene family in *Candida albicans* using 189 complete coding sequences retrieved from 23 genetically diverse strains across the species. Tlo genes conformed to the 3 major architectural groups (α/β/γ) previously defined in the genome reference strain but significantly differed in the degree of within-group diversity. One group, Tloβ, was always found at the same chromosome arm with strong sequence similarity among all strains. In contrast, diverse Tloα sequences have proliferated among chromosome arms. Tloγ genes formed 7 primary clades that included each of the previously identified Tloγ genes from the genome reference strain with 3 Tloγ genes always found on the same chromosome arm among strains. Architectural groups displayed regions of high conservation that resolved newly identified functional motifs, providing insight into potential regulatory mechanisms that distinguish groups. Thus, by resolving intraspecies subtelomeric gene variation, it is possible to identify previously unknown gene family complexity that may underpin adaptive functional variation.

## Introduction

Gene families are the result of repeated rounds of gene duplication that gives rise to similar or identical paralogs through errors in DNA replication, sister chromatid exchange, or whole genome duplication (Tilley and Birshtein 1985; Kellis *et al.* 2004; Mehta and Haber 2014; Reams and Roth 2015; Qiao *et al.* 2018). In most cases, one of the paralogs is inactivated by deleterious mutations following duplication, thereby restricting further evolutionary outcomes of paralogy (Cliften *et al.* 2006; Albalat and Cañestro 2016). However, duplicate genes that remain functional may retain the ancestral function, split the ancestral function or interaction networks between paralogs, or evolve specialized or novel functions (Hughes 1994; Wapinski *et al.* 2007; Des Marais and Rausher 2008; Innan and Kondrashov 2010). Retention of paralogs following repeated gene duplications can lead to the formation of a complex gene family whose members have the potential to diverge under divergent selective pressures or drift over evolutionary time.

Functional studies of gene family expansion have usually focused on paralog pairs in order to simplify inferences about selective pressures on genes following amplification (Kondrashov *et al.* 2002; Wagner 2002; Brunet *et al.* 2006; Cliften *et al.* 2006; Guan *et al.* 2007). Yeast species that have undergone whole genome duplication or regional mutations also make studies of many gene duplicates simultaneously convenient (Dietrich 2004; Kellis *et al.* 2004; Scannell, Butler, *et al.* 2007; Scannell, Frank, *et al.* 2007; Albertin and Marullo 2012). Studies of gene duplication demonstrated that functional outcomes are influenced by genomic context (Zhao and Boerwinkle 2002; Carreto *et al.* 2008; Zhu *et al.* 2014), gene dosage and protein complex formation (Aury *et al.* 2006; Veitia *et al.* 2008; Makino and McLysaght 2010), as well as by gene expression level (Aury *et al.* 2006; Conant and Wolfe 2006). However, the functional roles of individual paralogs from large gene families in fungi that expanded beyond a few copies remain largely unexplored, despite numerous developmentally and ecologically important gene family expansions (Brown *et al.* 2010; Floudas *et al.* 2012; Virágh *et al.* 2022).

Expanded gene families are often enriched in subtelomeric regions that are immediately adjacent to the telomeric repeats. Subtelomeres harbor a mixture of duplicated genes and repetitive sequences from fragmented mobile genetic elements (Corcoran *et al.* 1988; Riethman 2008; Kupiec 2014). In addition to copy number variation, subtelomeric genes are characterized by a rapid accumulation of mutations that can alter their expression, structure, or function (Brown *et al.* 2010). Frequent recombination, elevated mutation rates via acquisition of single nucleotide polymorphisms and insertions/deletions (indels), and the constant processes of gene duplication and disruption contribute to subtelomeres often being the most dynamic regions of the genome (Winzeler *et al.* 2003; Linardopoulou *et al.* 2005; Carreto *et al.* 2008; Kasuga *et al.* 2009; Anderson *et al.* 2015; Yue *et al.* 2017; Chen *et al.* 2018). Importantly, gene families that reside within the subtelomeres are often under selection from species-specific lifestyles (Mefford 2001; Dujon *et al.* 2004; Kyes *et al.* 2007; Linardopoulou *et al.* 2007; Brown *et al.* 2010; Chen *et al.* 2018; Otto *et al.* 2018). For example, the *MAL*, *MEL*, and *SUC* genes in Saccharomyces *cerevisiae* allow cells to utilize different carbon sources (maltose, melibiose, and sucrose, respectively), and fluctuate in copy number depending on the available growth substrate (Brown *et al.* 2010; Wenger *et al.* 2011; Dunn *et al.* 2012). Likewise, the opportunistic fungal pathogen *C. glabrata* encodes cell surface proteins termed *EPA* genes in their subtelomeres that facilitate adhesion to human epithelia in support of colonization and dissemination in the host (Mundy and Cormack 2009).

The expansion of several gene families involved in virulence traits distinguishes *Candida albicans*, the most clinically relevant *Candida* species, from closely related yeasts. Expansion of the *ALS*, *SAP*, and *LIP* gene families in *C. albicans* increases the available repertoire of adhesins, proteases, and lipases, respectively, which contribute to host colonization and tissue destruction (Magee *et al.* 1993; Hube *et al.* 2000; Hoyer 2001). The most dramatic gene expansion occurred within the telomere-associated (*TLO*) gene family, which are present in 14 copies in the *C. albicans* genome reference strain SC5314, 2 copies in the most closely related *C. dubliniensis* species, and a single copy within all other *Candida* species (Butler *et al.* 2009; Jackson *et al.* 2009). All but 1 *TLO* gene are found in the subtelomeres of the eight *C. albicans* chromosomes where they often reside as the ultimate or penultimate gene. The 14 *TLO* genes can be separated into 3 architectural groups (α, β, and γ) based on sequence variation that clusters toward the 3′ end of the gene (Van het Hoog *et al.* 2007; Anderson *et al.* 2012). *TLO* genes display high levels of sequence similarity. *TLO* paralogs have ∼97% nucleotide identity within a clade and 82% identity between clades when excluding indels (Van het Hoog *et al.* 2007; Anderson *et al.* 2012).

**Fig. 1.**
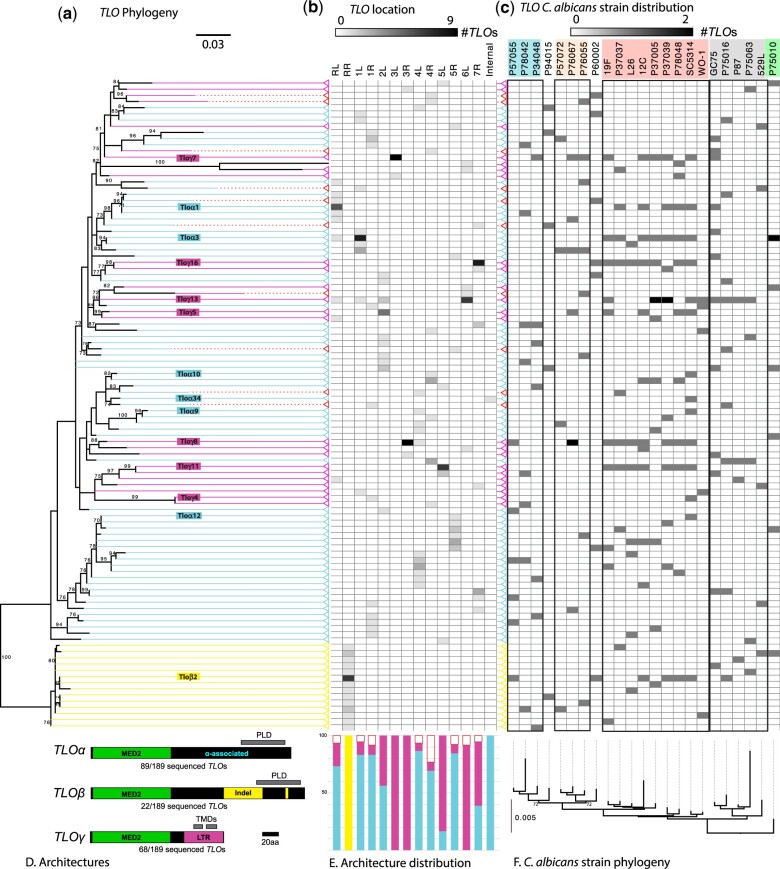
Tlo architectures across *Candida albicans* isolates. a) A maximum likelihood (ML) phylogeny of 189 translated *TLO* sequences was constructed under the JTTDCMut + G4 evolutionary model in IQTree. Identical sequences were collapsed into a single taxon prior to analysis. Support values are the percentage of 1000 IQTree UFBoot method. Terminals correspond to identical sequences with color indicating Tlo architectures (Tloα, solid cyan; Tloβ, solid yellow; Tloγ, solid pink; truncated, dashed red lines). b) The frequency of *TLO* sequences being found on each chromosome arm is indicated as a heat map. Internal denotes *TLO*α*34* from SC5314. c) The distribution of defined Tlos in the genome for each of the 23 *C. albicans* strains is indicated as a heat map. Strains are color coded by MLST clade as described in Hirakawa *et al.* (2015). d) Each Tlo architecture is indicated based on the presence or absence of sequence features color coded by group. LTR denotes long-terminal repeat and MED2 indicates a functional domain. Predicted PLD and TMDs are indicated with grey bars. e) The relative representation of Tlo architecture is indicated for each chromosome arm as a percentage of the total number of complete sequences. f) A whole genome phylogeny of 23 *C. albicans* strains. Clusters of genetically related strains are outlined in black boxes.

*TLO* genes encode a conserved N-terminal MED2 domain that facilitates their incorporation as stoichiometric components of the major transcriptional regulation complex Mediator (Zhang *et al.* 2012). Downstream of the MED2 domain is a gene-specific region of variable length followed by the 3′ portion of the gene that defines 3 *TLO* architectural types (α/β/γ, see Fig. 1d). *TLO*β*2* resides at the syntenic locus to *MED2* orthologs in other *Candida* species (Jackson *et al.* 2009) although *TLO*α group members appear to have given rise to *TLO*γ genes based on inferred mutational history (Anderson *et al.* 2012). The single *TLO*β group member contains two indels relative to *TLO*α group sequences, and *TLO*γ group members are defined by an LTR *rho* insertion that introduced a stop codon and truncated the coding sequence (Anderson *et al.* 2012). Recent diversification of these genes in *C. albicans* has resulted in variable *TLO* copy numbers among clinical isolates (Hirakawa *et al.* 2015), consistent with rapid gene loss/gain during in vitro passaging (Anderson *et al.* 2015).

Subtelomeric gene evolution in *C. albicans* has not been thoroughly explored at the individual sequence level because of the complexities in accurately resolving paralog gene structure and sequences from whole genome sequencing assemblies. Here, we obtained complete sequences of the subtelomeric *TLO* genes in 23 well characterized strains. *TLO* sequences provided evidence for complex evolutionary histories among groups in this single gene family. Sequenced genes conformed to 1 of the 3 previously defined architectural groups (α, β, γ) with the exception of a small number of truncated open reading frames. We further identified strong conservation of a prion-like domain (PLD) in a majority of Tloα and Tloβ sequences and 2 transmembrane regions in most Tloγ proteins. Surprisingly, phylogenetic analyses suggest that while Tloβ is monophyletic, Tloγ and truncated architectures may have emerged multiple times from the Tloα architecture. The degree of sequence divergence among groups varied significantly with high similarity among Tloβ sequences and high diversity among Tloα proteins. These evolutionary processes have resulted in diverse *TLO* repertoires among strains of *C. albicans* the functional consequences of which remain to be investigated.

## Materials and methods

### Amplification and sequencing of individual TLO paralogs

*Candida albicans* strains used in this study are listed in Supplementary Table 4. Overnight cultures of each *C. albicans* strain were grown overnight in 3 ml of liquid YPD medium on a rotary drum at 30°C. DNA was purified from these cultures using the MasterPure Yeast DNA Purification Kit (Epicenter/Lucigen). Purified DNA was used to amplify each *TLO* gene using a centromeric chromosome arm specific primer (ALO36-48 and ALO60) paired with a conserved *TLO* start site primer (ALO35) (Supplementary Fig. 1). Primer sequences are listed in Supplementary Table 5. The Accustart Taq DNA polymerase HIFI kit (Quantabio, USA) was used according to the manufacture’s instruction with the following cycling conditions: 1 cycle (3 min at 94°C), 35 cycles (20 s at 94°C, 30 s at 55°C, 1 min at 68°C), and 1 cycle (1 min at 68°C). Amplified products were examined on agarose gels by electrophoresis to confirm single, clean amplicons. Single-product amplification reactions were then purified using a magnetic bead approach (Berensmeier 2006). Purified DNA was sent for Sanger sequencing at the Genomics Shared Resource within the Ohio State University Comprehensive Cancer Center.

Sequencing was performed with primers oriented toward the *TLO* start site (ALO49-59 and ALO61) to sequence the amplified product (Supplementary Fig. 1). Chr4R was sequenced using the *TLO* start site primer. A minimum of 2 independent sequencing reactions were performed for each *TLO* amplicon. The conserved start site primer was used to resolve any ambiguous sequence near the start codon.

### Phylogenetic reconstructions

Consensus *TLO* nucleotide sequences were translated into amino acid sequences using the CUG fungal translation table (alternative yeast nuclear code—translation table 12). Sorted alignments were built from the 189 consensus Tlo sequences using MAFFT v.7 (Katoh and Standley 2013). Sequences were divided by group architecture (α, β, γ); where *TLO*β-like sequences contained 1 large and 1 small insertion, *TLO*γ-like sequences were interrupted by an LTR, and *TLO*α-like sequences contained neither of these events. Domain extraction was conducted for all full length sequences and also in isolation of the MED2 domain using HMMER v3.3.2 (Eddy 2011). ModelFinder (Kalyaanamoorthy *et al.* 2017) within IQ-TREE (Nguyen *et al.* 2015) was used for evolutionary model testing. Maximum likelihood phylogenies were run with 1000 bootstrap replicates within IQ-TREE using the ultrafast bootstrap (UFBoot) (Hoang *et al.* 2018) method under the best evolutionary model. Bootstrapped trees were then exported as Newick trees for visualization.

### Constraint analyses

Monophyletic node architectures (Supplementary Table 6) were constructed in Mesquite v3.70 (http://www.mesquiteproject.org, last accessed 05/01/2021). Constrained topologies generated in IQ-TREE were compared by the Direct Computation with Mutabilities revised JTT model (JTTDCmut) with a gamma of four categories (Kosiol and Goldman 2005; Nguyen *et al.* 2015). Statistical analysis of these constraints is reported in Table 1.

**Table 1.** Results of constraint analyses.

Constraint	*P*-value^a^	LogL
None (maximal likelihood tree)	0.693	−2983.985
Alpha monophyletic	**0.015**	−3054.839
Beta monophyletic	0.583	−2986.888
Gamma monophyletic	0.134	−3020.201
Beta gamma both monophyletic	0.134	−3020.201
Truncated monophyletic	**0.021**	−3030.799
Truncated free, architectures monophyletic	0.086	−3024.149
Group architectures each monophyletic	**0.004**	−3069.540

^a^ *P*-AU reported: *P*-value of approximately unbiased (AU) test (Shimodaira 2002). Significant differences (*P*-value < 0.05) between the best unconstrained and constrained topologies are indicated by bolding.

### PLD identification

PrionW (Zambrano *et al.* 2015) was used to predict PLDs based on an amyloid core and predicted pWALTZ score. PLAAC analysis (Lancaster *et al.* 2014) was used to identify the strength to which PLD calls conformed to the canonical yeast PLD architecture based on hidden Markov modeling (Supplementary Fig. 4).

### Identification of predicted transmembrane domains in Tlog group members

The Protein Homology/analogY Recognition Engine V 2.0 (Phyre2) web portal (Kelley *et al.* 2015) was used on “Expert Mode” to generate structural predictions for the 68 Tloγ group members from *C. albicans*. A FASTA file containing the 68 Tloγ protein amino acid sequences was edited to remove any gaps and non-letter characters before it was submitted to the “Batch Processing” portal of Phyre2. Default parameters were used when applicable. Domain regions were then mapped back to the Tloγ group member MAFFT alignment for visualization.

### Data visualization

Data visualization was conducted using R version 3.6.3. Bar charts were generated using Microsoft Excel. Newick format trees were visualized using FigTree v1.4.4 (http://tree.bio.ed.ac.uk/software/figtree/, last accessed 08/01/2021). Sequence conservation and consensus were visualized using Jalview (Waterhouse *et al.* 2009). Amino acid sequence logos were visualized using the R package “ggseqlogo” (Wagih 2017) and colored using the default ClustalX coloring scheme (Thompson *et al.* 1994).

## Results

Short-read sequencing of 23 *C. albicans* clinical isolates failed to accurately incorporate sequence variants into subtelomeric genes known to be present in the resulting assemblies (Hirakawa *et al.* 2015). These isolates capture much of the diversity present in *C. albicans* as they originate from different geographic regions, body sites of isolation, and a range of clades within the species (Butler *et al.* 2009; Hirakawa *et al.* 2015; Cuomo *et al.* 2019). To determine intraspecies variation in *C. albicans* subtelomeric genes, we employed a chromosome-arm specific amplification and sequencing strategy that is capable of identifying any *TLO* gene present on a given chromosome arm through the use of centromeric chromosome arm-specific primers in combination with a primer that binds to a conserved sequence at the *TLO* start codon (Supplementary Fig. 1). Resolved full length sequences facilitated characterization of gene architecture and mapping of structural and location data to a comprehensive gene phylogeny. This enabled the inference of trends in *TLO* molecular evolution and possible key events in the diversification of *TLO* genes across *C. albicans*.

### *Candida albicans* has a positionally and architecturally *diverse TLO* repertoire

*TLO*-specific amplification was performed for both subtelomeres of all 8 *C. albicans* chromosomes in the 23 genetic backgrounds. Each of the resulting 299 amplicons were Sanger sequenced bidirectionally to produce 189 total full *TLO* gene sequences, representing between 4 and 14 products for each isolate (Fig. 1, a and b). Consistent with the genome reference strain SC5314, the right arm of chromosome 2 (Chr2R), Chr6R, and Chr7L did not yield any amplification products for any strain, indicating these chromosome arms do not encode *TLO* genes in *C. albicans*. All Tlo sequences contained an intact MED2 domain and were subsequently sorted into 3 *TLO* architectural groups based on similarity within an MAFFT alignment of the inferred protein sequences (Fig. 1d). In total, 89 sequences conformed to the Tloα group gene architecture, 22 sequences to the Tloβ group, and 68 sequences to the Tloγ group. Conservation of specific Tlo sequences among related strains was evident in some cases but only in a minority of sequences (Fig. 1c). The number of amplified *TLO* genes nor their relative representation among the three groups (α/β/γ) correlated with multilocus sequence type (MLST) clade designations (Supplementary Table 1). Additionally, nonsense mutations disrupted 10 additional *TLO* sequences that are predicted to encode a complete MED2 domain but very little C-terminal peptide sequence and, therefore, did not conform to the α/β/γ group architecture.

We obtained good representation of *TLO* sequences for most chromosome arms, which revealed a clear pattern of *TLO* gene representation. *TLO*β*2* genes were consistently recovered only from ChrRR. The other chromosome arms contained either only *TLO*γ sequences or a mix of *TLO*α and *TLO*γ genes. Extensive *TLO*α/γ group swapping was observed on Chr2L and Chr7R, while the only intact loci on Chr3L, Chr3R, and Chr6L encoded *TLO*γ group members (Fig. 1e).

Transcription factors (TFs) containing PLDs can form phase-separated condensates in *C. albicans* that regulate cell identity (Frazer *et al.* 2020). We speculated similar molecular mechanisms may regulate transcriptional activators, including Tlo proteins. Indeed, 91 of 189 Tlos contained a previously unidentified putative PLD (Fig. 1d;Supplementary Table 2). PLDs were restricted to Tloα and Tloβ group proteins, although some Tloα and Tloβ sequences lacked a recognizable PLD (e.g. ChrRR in L26, ChrRR in P37005; Supplementary Fig. 2).

Immunoprecipitation and mass spectrometry previously confirmed Tloα and Tloβ proteins associate with Mediator as predicted Med2 orthologs but failed to identify Mediator-bound Tloγ proteins (Zhang *et al.* 2012). Scanning the Tloγ sequences for unknown motifs uncovered 2 putative transmembrane domains (TMDs) in the C-terminal 50 amino acids (AA) in all but 2 Tloγ group members (Fig. 1d). Specifically, the Tloγ sequence on Chr4R of P78042 contained only one predicted TM region, and none were predicted in Tloγ4, a previously described Tloγ truncation on Chr1R of SC5314 (Anderson *et al.* 2012). The 2 predicted transmembrane helices are separated by a short 3 AA cytoplasmic loop that would place most of the Tloγ protein, including the Med2 domain, on the internal face of the mitochondrion.

### The *MED2* domain is highly conserved across *TLO* genes

The N-terminal MED2 domain defines *TLO* genes as Med2 homologs that are incorporated as subunits of the larger Mediator complex (Yin and Wang 2014; Plaschka *et al.* 2016). A HMMER search for defined protein motifs recovered the conserved MED2 domain in all 189 Tlo sequences and identified 90 homologous amino acid positions that are present in all sequences. Maximum likelihood phylogenetic analysis of the Tlo MED2 domain alignment did not recover distinct α/β/γ clades previously inferred using structural architecture (Supplementary Fig. 2). Minimal branch lengths separated most Tlo MED2 sequences with the major exception of the *TLO*s on Chr6L in P37037 and P78048. These 2 MED2 sequences are strongly separated from the rest of the phylogeny by amino acid variants that begin two-thirds of the way through the MED2 domain (Chr6L in P37037; 71–90 AA, Chr6L in P78048; 62–90 AA).

### Monophyly is only strongly supported for the *TLO*β architecture

We built an MAFFT alignment of the homologous sequences among all *TLO* genes and further refined the output manually. Application of maximum likelihood to infer evolutionary relationships among architectures supported monophyly of Tloβ genes, whereas Tloα and Tloγ sequences were intermixed (Fig. 1a). To test for monophyly of each Tlo architecture, constraint analyses (Table 1) were performed on each Tlo architecture independently and in all possible pairings. Constrained analyses that forced each individual architecture into a single node using the full dataset rejected the monophyly of the Tloα [approximately unbiased (AU) test; *P* = 0.015) and truncated architectures (AU test; *P* = 0.021). Monophyly was not rejected for the *Tlo*β and Tloγ architectures using the full dataset, but monophyly of Tloγ was rejected in an alignment that excluded the truncated sequences (AU test; *P* = 2.09E−3). Assuming that *TLO*β is the ancestral architecture based on shared synteny between the ChrRR locus and chromosomes encoding *MED2* homologs in other *Candida* species (Jackson *et al.* 2009), these results suggest that *TLOα* genes likely arose more than once by duplication of *TLO*β, and *TLO*γ architectures arose from *TLOα*, possibly only once and subsequently expanded. Truncated architectures arose from both *TLO*α and *TLO*γ sequences.

### *TLO*β sequence architectures are conserved

**Fig. 2.**
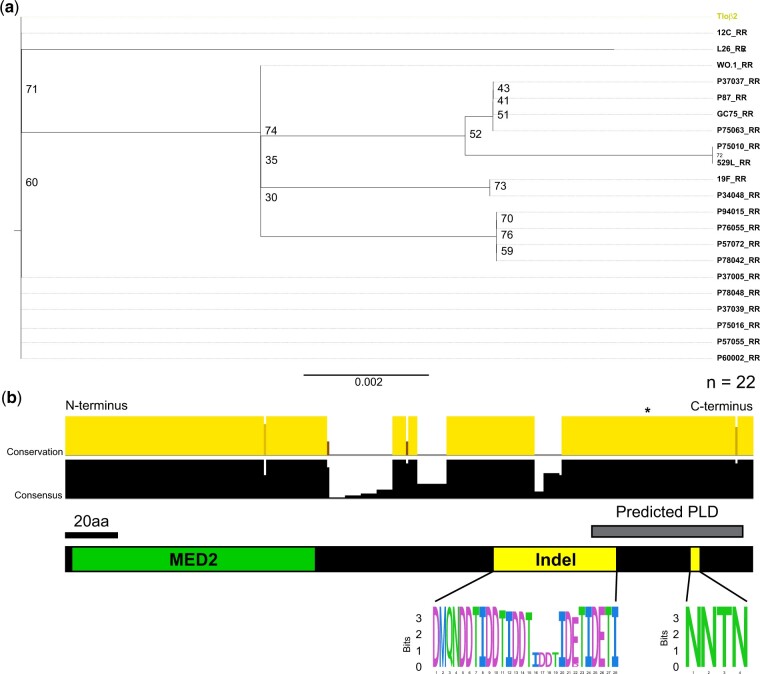
*TLOβ* group genes display little variation. a) The 22 Tloβ group sequences were assessed for evolutionary relationships using maximum likelihood with JTT + G evolutionary models and 1000 UFBoot replicates. *TLO* sequences are reported as “Patient Isolate_Chromosome Arm.” Tlo sequence from SC5314 is colored. b) The canonical *TLO*β group structure is cartooned, where the 2 insertion events have been isolated as a sequence logo. Letters represent individual AA where the height signifies site representation. AA within the sequence logo are colored using the default ClustalX coloring.

Twenty-two sequences were identified as *TLO*β architectures based, in large part, on the presence of 2 defining indels toward the 3′ end of the gene when compared with other *TLO* architectures. Analysis of 189 Tloβ homologous positions revealed relatively little divergence among these sequences (Fig. 2a). Only 3 positions encoded amino acid variants in the full alignment of all 22 Tloβ sequences (AA 117, 152, and 298 in the full alignment). Most variation in Tloβ sequences involved expansion or contraction of one of the defining indels rather than amino acid substitutions. Sequence variants in Tloβ genes clustered immediately after the MED2 domain in the first of 2 Tloβ-defining indels and corresponded to copy number variation of a tandem repeat that codes for a “TIDD/E” amino acid sequence (Fig. 2b). Eight Tloβ sequences contained 1 or 2 fewer “TIDD/E” amino acid repeats compared with the SC5314 reference genome. The Tloβ sequence in isolate L26 contained a nonsense mutation at amino acid position 209, shortening this Tloβ protein by 63 AA but did not interrupt either the MED2 domain or the *TLO*β-specific indels.

### *TLO*α genes are highly diversified

**Fig. 3.**
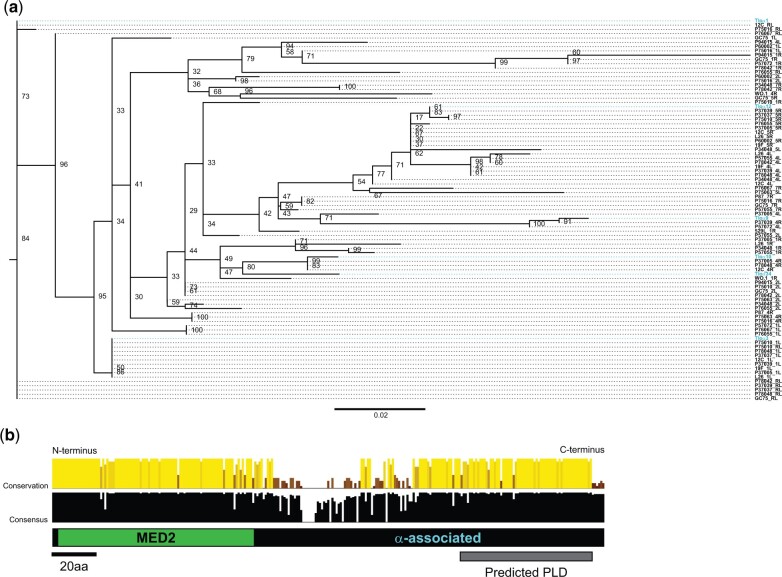
Expansive sequence variation among Tloα. a) Phylogenetic reconstruction as described in Fig. 2 was conducted for the 89 Tloα group sequences and the resultant tree was visualized with FigTree. Tlo sequences are reported as “Patient Isolate_Chromosome Arm.” Tlo sequences from SC5314 are colored. b) The canonical Tloα group structure is cartooned with the Med2 and PLD indicated. Conservation and consensus at each position are plotted at each position for all 89 Tloa sequences based on alignment using MAFFT. Bar height and strength of color signifies the strength at each position.

Radiating sequence diversity was present among *TLO*α members with most genes encoding a unique sequence relative to all other group members (Fig. 3a). To identify other conserved functional domains in Tloα genes, we inferred the consensus alignment of all Tloα sequences. Two regions displayed high conservation in the alignment: the N-terminal MED2 domain and a second region covering the putative C-terminal PLD (Fig. 3b). Truncation of four Tloα sequences by nonsense mutations occurs immediately downstream of the predicted PLD, but these genes still clustered with Tloα sequences.

### *TLO*γ sequences cluster around gene-specific clades

**Fig. 4.**
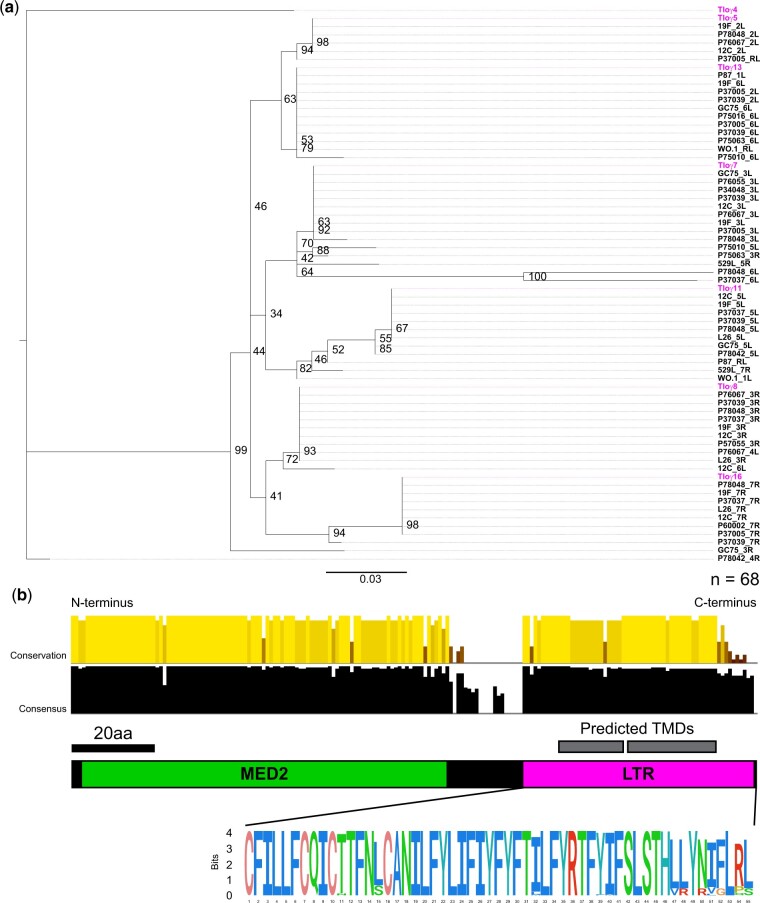
*TLOγ* group phylogenetic organization reveals gene clades. a) Phylogenetic reconstruction as described in Fig. 2 was conducted for the 68 Tloγ group sequences and the resultant tree was visualized with FigTree. Tlo sequences are reported as “Patient Isolate_Chromosome Arm.” Tlo sequences from SC5314 are colored. b) The conserved Tloγ group structure has been cartooned, where the LTR has been isolated as a sequence logo. Letters represent individual AA where the height signifies site representation. AA within the sequence logo are colored using the default ClustalX coloring.

The remaining 68 sequences in the dataset are truncated in an identical location by a single rho LTR 3′ insertion that defines the *TLO*γ architecture. The conserved sequence of the LTR insertion includes the 2 predicted TMDs. Altogether, 129 homologous sites were present in a Tloγ alignment that included 90 sites in the MED2 domain. The Tloγ-only phylogeny had moderate to strong bootstrap support at terminal nodes that contained highly similar sequences to single SC5314 Tloγ genes (Fig. 4a). Removal of two truncated *TLO*γ sequences (*TLO*γ*4* and Chr4R in P78042) increased the total number of informative sites from 129 to 156 but did not significantly alter the phylogenetic relationships among the Tloγ sequences.

Two-thirds of the Tloγ protein alignment (122/183 sites) is identical across sequences. Although the MED2 domain was expected to be conserved, the sequence within the Tloγ-defining *rho* LTR insertion was also surprisingly conserved (36/53 identical sites, Fig. 4b). Nine of the 17 variant sites in the LTR insertion are due to Tloγ16-like sequences that encode a distinct C-terminal nine amino acid peptide (VRYRVGLPS) with no notable similarity to other *C. albicans* genes.

*TLO*α-like sequences remain downstream of the rho LTR insertion that define the *TLO*γ architecture (Anderson *et al.* 2012). These sequences in SC5314 retain strong similarity to one another and the C-terminal end of Tloα proteins (Supplementary Fig. 3a). Inclusion of these sequences in the Tlo phylogeny did not significantly alter the topology of the tree and interspersed placement of Tloα and Tloγ sequences (Supplementary Fig. 3b).

### Gene disrupting mutations do not show any clear patterns

Ten *TLO* genes contained ORF-disrupting mutations that significantly truncated the coding sequence. The *TLO* located on ChrRL in P60002 experienced a frameshift due to a single nucleotide insertion, while nonsense mutations disrupted all other *TLO* genes. Most of the genes contain a premature stop between 130 and 154 AA, shortly after the MED2 domain, where sequence conservation immediately declines at AA92 in the alignment (Supplementary Fig. 3). It was possible to determine the architecture prior to truncation for each of these sequences, and this architecture is consistent with placement in the *TLO* phylogeny (Fig. 1; Supplementary Table 3). The *TLO*s on Chr4R in P76055, P60002, and GC75 share a 34 amino acid C-terminal peptide with no similarity to any other *C. albicans* protein that we hypothesize is a result of Chr4R sequence divergence following truncation.

## Discussion

Interrogation of expanded gene families often relies exclusively on variation present in the genome reference strain without considering additional intraspecies sequence diversity and is especially true for comparison of paralogs in subtelomeric gene families. Genetic variation in the *C. albicans TLO* subtelomeric gene family among 23 clinical strains expands our understanding of the of evolutionary processes shaping the gene repertoire during gene family expansion. Expansion and differentiation of the *TLO* family into 3 groups has resulted in distinct sequence conservation outcomes, ranging from strong conservation to diversification. Conserved segments of each *TLO* group alignment highlight previously overlooked functional domains that may contribute to functional diversification among paralogs. Together, this work reveals the balance between gene sequence diversification and novel functional motif conservation during subtelomeric gene family evolution that can confer unique attributes to gene subsets.

Expansion of the *C. albicans TLO*s likely occurred through multiple independent events. Only the Tloβ architectural group is clearly monophyletic in *C. albicans* and may reflect its position as the ancestral *TLO* structure. The conserved position of *TLO*β*2* on ChrRR and the synteny of the position with *MED2* orthologs in other *Candida* species (Jackson *et al.* 2009) raises the hypothesis that it may maintain the ancestral function. In support of this, both *TLO*β*2* and the syntenic *Candida dubliniensis TLO1* contribute to filamentation (Haran *et al.* 2014; Uppuluri *et al.* 2018). The *TLO*α group appears then to have emerged multiple independent times in the *C. albicans* lineage via loss of the two indels that distinguish these architectural groups. If the TLO phylogeny is correct, it is most parsimonious to infer that the insertion of the rho LTR into the *TLO*α sequence to produce the *TLO*γ architecture occurred multiple times. However, the common insertional position into *TLO*α by the same retroelement type that resulted in *TLO*γ and constraint analysis suggests the possibility of a single insertion event or this consistency could be the result of a region particularly vulnerable to disruption.

Most surprising from our analysis was that different *TLO* groups within the single expanded gene family have experienced disparate modes of sequence diversification. At one extreme, monophyletic Tloβ sequences have undergone very little sequence diversification despite our hypothesis that they represent the root of the *TLO* expansion. At the other extreme, Tloα proteins have undergone less constrained evolution and explore a wide swath of sequence space (Povolotskaya and Kondrashov 2010). This could reflect a longer amount of time for evolution to act on individual homologs or altered selective pressures on this gene architecture, which may no longer maintain the ancestral function. Lastly, the *TLO*γ sequences fall between these opposing extremes with clearly delineated clades that correspond to the *TLO*γ genes present in SC5314. The most parsimonious explanation for the *TLO*γ architecture is that *TLO*γ paralogs have come under purifying selection following sequence diversification that occurred early in the diversification of *C. albicans* since many unrelated strains retain the same sequence in the same locus. That 2 paralogous genes, the ancestral *TLO*α and *TLO*β have such different diversification patterns highlights the potential of gene family diversification to facilitate species adaptation. Organismal benefit may be derived from the alternative protein–protein interactions and transcriptional states conferred by incorporation of unique *TLO* structures and sequences into the transcriptional regulatory complex Mediator. Formation of alternate regulons by production of different Mediator types may operate as a bet hedging mechanism in the same cell or among cells in a population. Indeed, a vast excess of Tlo to Mediator in *C. albicans* (Zhang *et al.* 2012; Haran *et al.* 2014; Liu *et al.* 2016) could allow *TLO* genes to take unconstrained or additional evolutionary strategies independent of their orthologous role as Mediator components.

The molecular role of a PLD in regulating availability of Mediator subunits has emerged as a common post-transcriptional regulatory mechanism (Zhu *et al.* 2015; Batlle *et al.* 2021). The PLD predicted in the majority of *TLO*α and *TLO*β paralogs contains a characteristic amyloid core required for phase transition of master regulators that define cell states in eukaryotic species (Hnisz *et al.* 2017). Recent work demonstrated similar mechanisms regulate *C. albicans* TFs that govern transition between the white and opaque cell states (Frazer *et al.* 2020). Given that *TLO*α and *TLO*β sequences function interchangeably as the Med2 subunit of Mediator (Zhang *et al.* 2012, 2013), it is tempting to speculate that Tlos form liquid-like droplets to sequester excess Tlo from Mediator or with Mediator itself. Sequestration of Tlo may alter general transcriptional activity of Mediator or change the patterns of RNA Polymerase II (PolII) recruitment to promoters by increasing association of Mediator with a subset of available Tlos (Haran *et al.* 2014).

Alignment of the Tloγ revealed the strong conservation over the LTR insertion that defines this *TLO* group. Conservation of this insertion indicated an embedded functional domain that led to the identification of TMDs that may anchor Tloγ proteins in the outer mitochondrial membrane or internally in cristae. How their putative membrane association contributes to their ascribed function in Mediator is unclear since this complex is expected to require free diffusion and may suggest a mitochondrial function completely independent of its canonical role in Mediator (Mamouei *et al.* 2021).

Truncation of 10 genes by nonsense mutations appears to have resulted in MED2 domains with little downstream sequence. *MED2* homologs in ascomycetes tend to contain an N-terminal MED2 domain followed by an extended C-terminal tail. Yet, the Med29 metazoan counterpart to fungal Med2 lacks the C-terminal extension in its role in the Mediator tail (Rengachari *et al.* 2021). Retention of the MED2 domain should allow association with Mediator, but how this affects recruitment of PolII and gene expression when lacking the C-terminal end to interact with TFs is unclear.

This investigation reinforced previous work showing frequent “movement” of *TLO* paralogs between chromosome arms (Anderson *et al.* 2015). Interestingly, the chromosome arms containing *TLO*α group sequences also always contain *TLO*γ sequences in other isolates, suggesting that the more recently emerged *TLO*γ genes may be more flexible in occupying various chromosomal position compared with *TLO*α genes. This is consistent with the unidirectional invasion and replacement of *TLO*α by *TLO*γ genes during passaging experiments with SC5314 (Anderson *et al.* 2015). While an eventual complete replacement of *TLO*α group members by *TLO*γ genes may be expected in this framework, a divergent function of *TLO*γ genes from *TLO*α paralogs may restrict their abundance among the *TLO* repertoire. Lastly, the placement of *TLO*β is consistently on ChrRR in each sequenced isolate may have resulted from the absence of a telomere recombination element (TRE) adjacent to *TLO*β*2*, previously noted in SC5314 (Freire-Benéitez *et al.* 2016). Disruption of a TRE reduced rates of loss of heterozygosity on single chromosome arms and may similarly reduce interchromosomal recombination and gene “movement” to other chromosome arms when absent.

Altogether, this work demonstrates that subtelomeric gene family diversity is likely significantly underrepresented when using a single genome reference strain for eukaryotic species. As a result, current perspectives of genome evolution in functional subtelomeric sequences may be incomplete or skewed based on the limited data available in a single isolate. As seen for *C. albicans TLO* genes, expansion to include a strain collection revealed sequence diversification and the evolutionary histories of individual or groups of genes that were otherwise hidden.

## Supplementary Material

jkac283_Supplemental_Material_Legends

jkac283_Supplementary_Figure_S1

jkac283_Supplementary_Figure_S2

jkac283_Supplementary_Figure_S3

jkac283_Supplementary_Figure_S4

jkac283_Supplementary_Figure_S5

jkac283_Supplementary_Table_S1

jkac283_Supplementary_Table_S2

jkac283_Supplementary_Table_S3

jkac283_Supplementary_Table_S4

jkac283_Supplementary_Table_S5

jkac283_Supplementary_Table_S6

## Data Availability

All sequences used as part of this manuscript have been deposited in GenBank under accession numbers OP580297–OP580471, https://www.ncbi.nlm.nih.gov/popset/?term=OP580297%3AOP580471. Supplemental material is available at G3 online.
