# Supplementary material for: Architectural groups of a subtelomeric gene family evolve along distinct paths in Candida albicans
Source: G3 (Bethesda). 2022 Oct 21;12(12):jkac283. doi: 10.1093/g3journal/jkac283 (PMC9713401; doi:10.1093/g3journal/jkac283)
Supplement: jkac283_Supplemental_Material_Legends [file jkac283_supplemental_material_legends.docx]

**SUPPLEMENTAL FIGURE LEGENDS**

**Figure S1. *TLO* sequencing strategy.** The strategy used to amplify *TLO* gene sequences from specific chromosome arms has been cartooned where relative locations of each of the primer sets used are specified. Complete primer list can be found in Supplemental Table 2.

**Figure S2. The *TLO* MED2 domain is evolving independent of the C-terminal sequence architecture. (A)** Phylogenetic reconstruction of the HMMER extracted MED2 domain was conducted on the 189 sequenced *TLO*s using 90 homologous sites. Maximum likelihood phylogenies were constructed using JTT+G evolutionary models with 1000 ultrafast bootstrap (UFBoot) replicates. Coloration was assigned by *TLO* group, where cyan represents *TLO*α members, yellow represents *TLO*β members, magenta represents *TLO*γ members, and red represents Tlo sequences outside of these three groups. Grey coloration indicates the presence of a prion-like domain (PLD). *TLO* sequences are reported as “Patient Isolate_Chromosome Arm.” Bootstrap values greater than 0.5 are shown at their respective nodes.

**Figure S3. Conserved Tloα sequences lie downstream of *TLO*γ genes. (A)** The region downstream of the architecture-defining *TLO*γ LTR insertion was translated for SC5314 and aligned with Tloα sequences using MAFFT. The conservation, quality, consensus, and occupancy at each site is given below. The intensity of blue shading indicates the similarity to the consensus residue at that position. (**B)** The evolutionary relationship of all 189 Tlo sequences and the reconstructed “Tloγ” genes prior to LTR disruption were assessed using maximum likelihood with JTT+G evolutionary models and 1000 ultrafast bootstrap (UFBoot) replicates. *TLO* sequences are reported as “Patient Isolate_Chromosome Arm.”

**Figure S4. Truncated Tlo sequences are disrupted at the C-terminus.** The ten truncated Tlo sequences were aligned using MAFFT and the conservation and consensus are indicated in each line where bar height and strength of color signifiy the strength at each position.

**Figure S5. Graphical representation of putative prion-like domains in Tlo proteins.** The results of PLAAC analysis for Tloα12 and Tloβ2 from the SC5314 genetic background are displayed.

**Table S1. *TLO* sequence architectures are not defined by their MLST genotype group.**

**Table S2. *TLO*α and *TLO*β group architectures contain PLD signatures in the 3’ architecture-specific region.**

**Table S3. Assignment of sequences downstream of ORF-disrupting mutations.**

**Table S4. Strains used in this study.**

**Table S5. Oligonucleotides used in this study.**

**Table S6. Constraint analysis by architecture and chromosomal position.**
