# Supplementary figures and images for: Architectural groups of a subtelomeric gene family evolve along distinct paths in Candida albicans
Source: G3 (Bethesda). 2022 Oct 21;12(12):jkac283. doi: 10.1093/g3journal/jkac283 (PMC9713401; doi:10.1093/g3journal/jkac283)

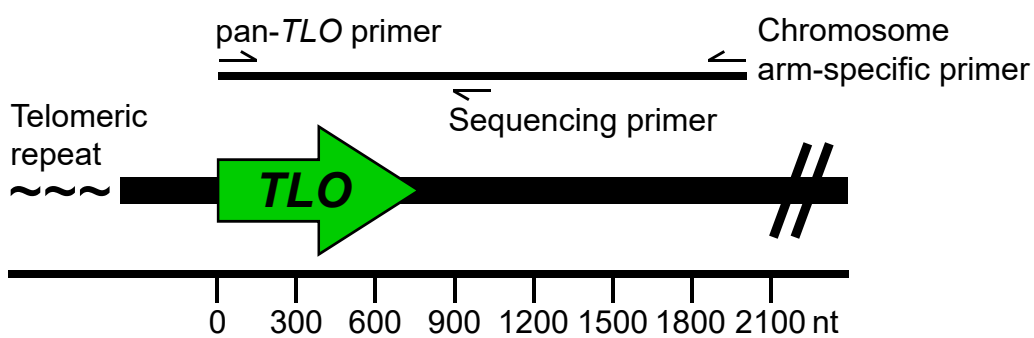

Supplement: jkac283_Supplementary_Figure_S1 [file jkac283_supplementary_figure_s1.pdf]

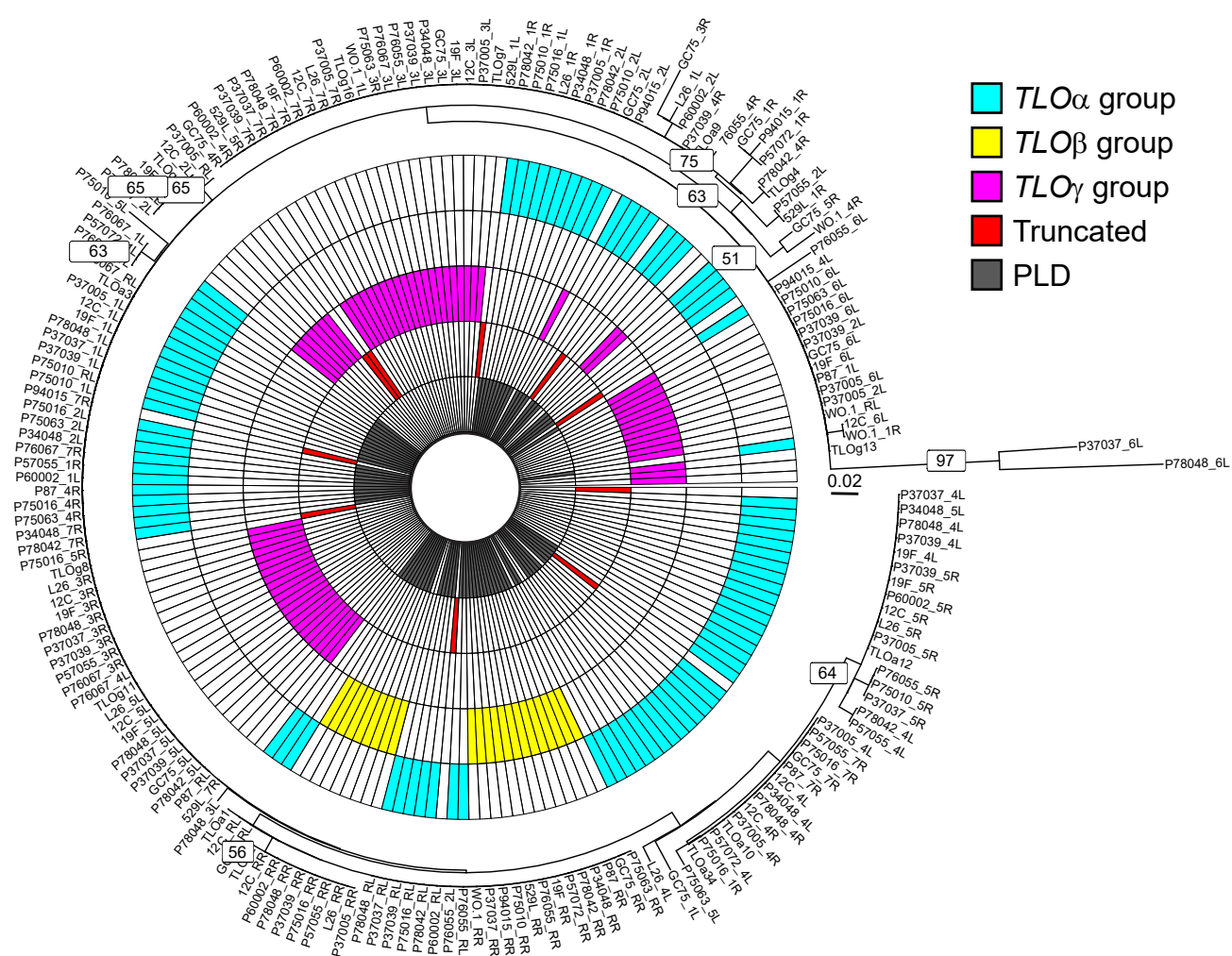

Supplement: jkac283_Supplementary_Figure_S2 [file jkac283_supplementary_figure_s2.pdf]

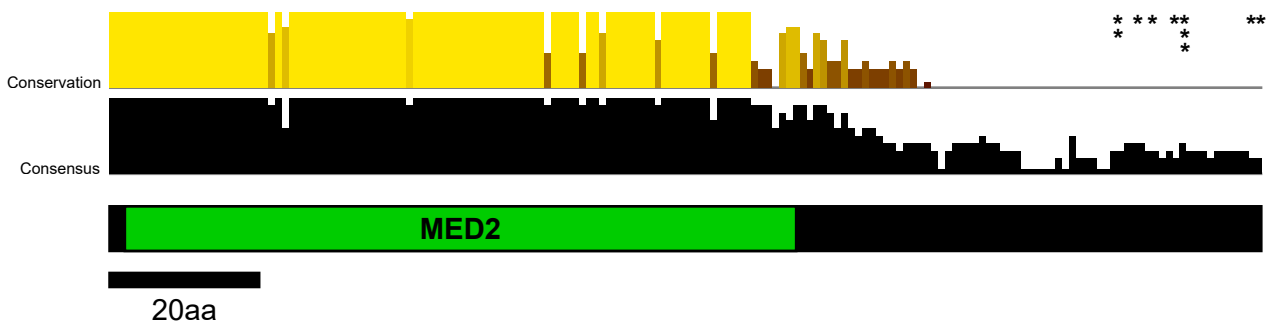

Supplement: jkac283_Supplementary_Figure_S4 [file jkac283_supplementary_figure_s4.pdf]

CaTlo $\alpha$ 1 2

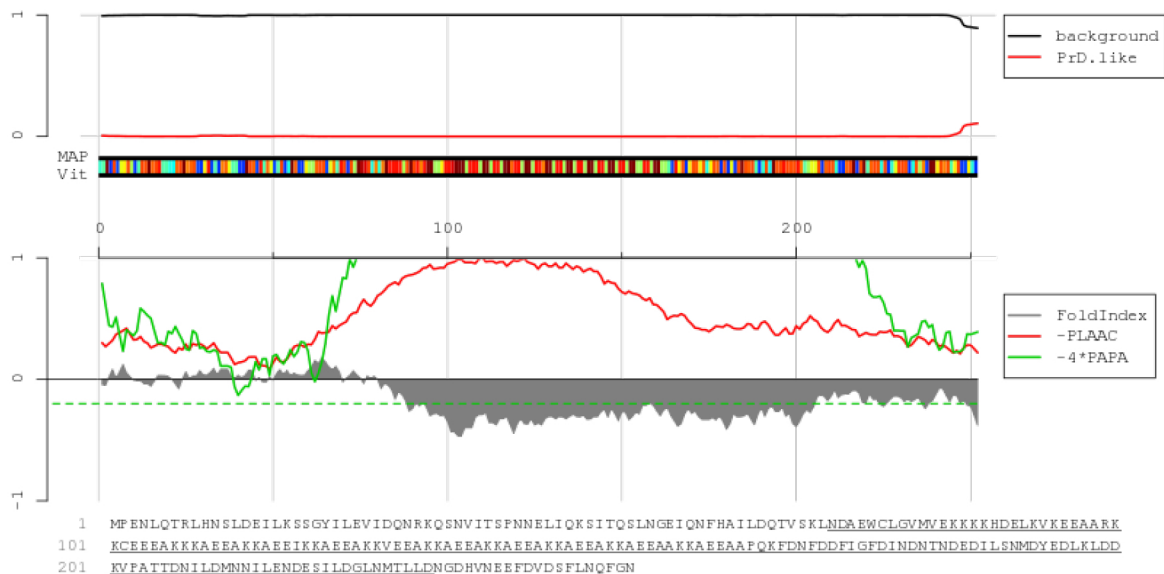

CaTloβ2

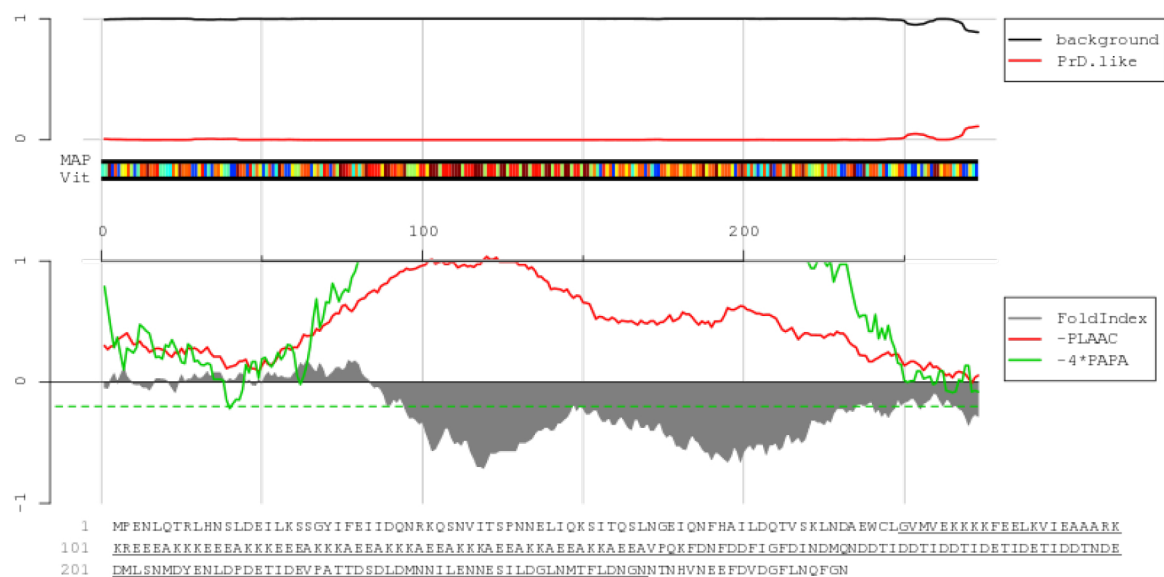

Supplement: jkac283_Supplementary_Figure_S5 [file jkac283_supplementary_figure_s5.pdf]
