# Supplementary material for: Architectural groups of a subtelomeric gene family evolve along distinct paths in Candida albicans
Source: G3 (Bethesda). 2022 Oct 21;12(12):jkac283. doi: 10.1093/g3journal/jkac283 (PMC9713401; doi:10.1093/g3journal/jkac283)
Supplement: jkac283_Supplementary_Figure_S3 [file jkac283_supplementary_figure_s3.pdf]

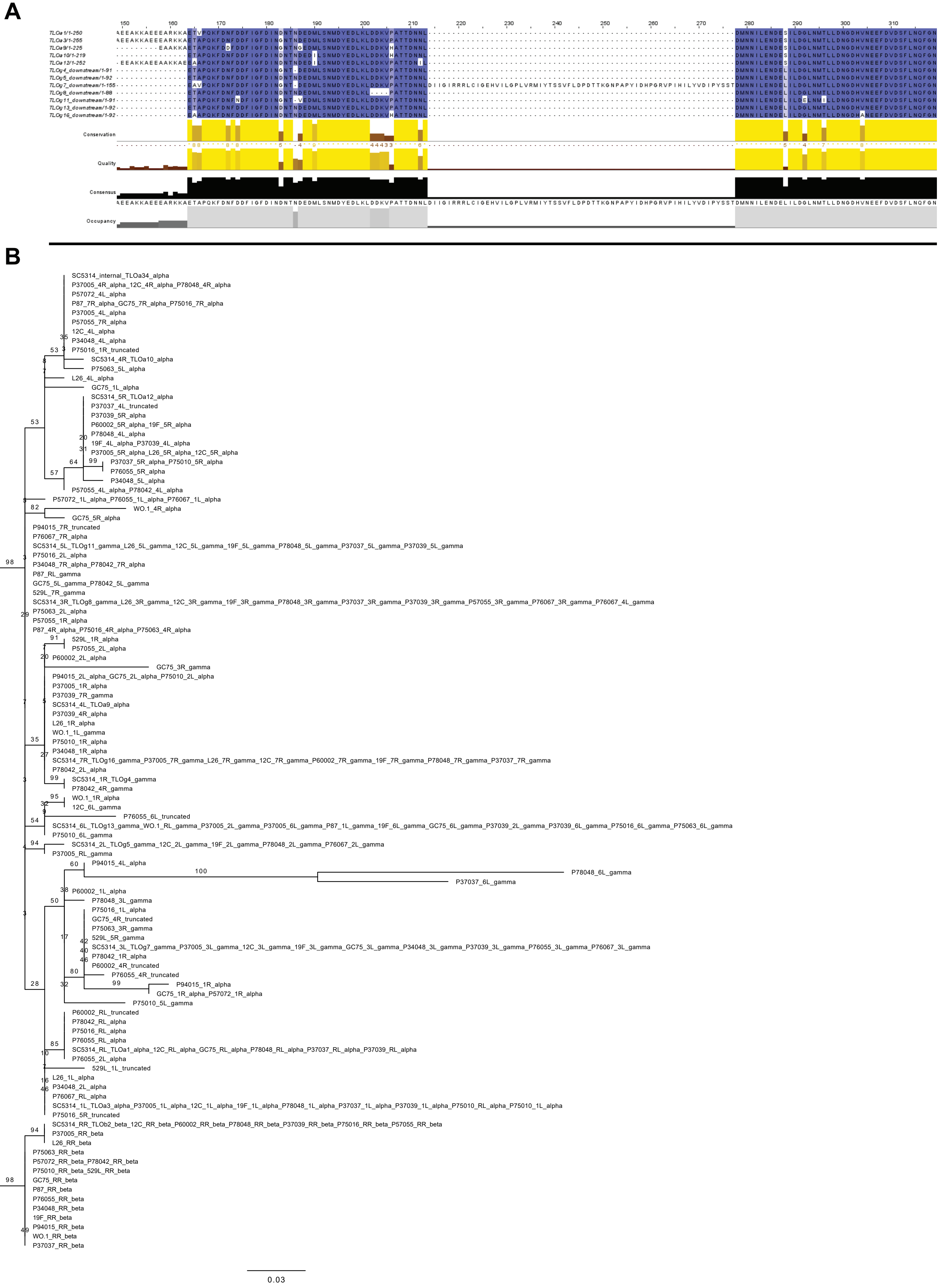

A
TLOa1/1-250
TLOa3/1-255
TLOa9/1-225
TLOa10/1-219
TLOa12/1-252
TLOg4_downstream/1-91
TLOg5_downstream/1-92
TLOg7_downstream/1-155
TLOg8_downstream/1-88
TLOg11_downstream/1-91
TLOg13_downstream/1-92
TLOg16_downstream/1-92
Conservation
Quality
Consensus
Occupancy
B
SC5314_internal_TLOa34_alpha
P37005_4R_alpha_12C_4R_alpha_P78048_4R_alpha
P57072_4L_alpha
P87_7R_alpha_GC75_7R_alpha_P75016_7R_alpha
P37005_4L_alpha
P57055_7R_alpha
12C_4L_alpha
P34048_4L_alpha
P75016_1R_truncated
SC5314_4R_TLOa10_alpha
P75063_5L_alpha
L26_4L_alpha
GC75_1L_alpha
SC5314_5R_TLOa12_alpha
P37037_4L_truncated
P37039_5R_alpha
P60002_5R_alpha_19F_5R_alpha
P78048_4L_alpha
19F_4L_alpha_P37039_4L_alpha
P37005_5R_alpha_L26_5R_alpha_12C_5R_alpha
P37037_5R_alpha_P75010_5R_alpha
P76055_5R_alpha
P34048_5L_alpha
P57055_4L_alpha_P78042_4L_alpha
P57072_1L_alpha_P76055_1L_alpha_P76067_1L_alpha
WO.1_4R_alpha
GC75_5R_alpha
P94015_7R_truncated
P76067_7R_alpha
SC5314_5L_TLOg11_gamma_L26_5L_gamma_12C_5L_gamma_19F_5L_gamma_P78048_5L_gamma_P37037_5L_gamma_P37039_5L_gamma
P75016_2L_alpha
P34048_7R_alpha_P78042_7R_alpha
P87_RL_gamma
GC75_5L_gamma_P78042_5L_gamma
529L_7R_gamma
SC5314_3R_TLOg8_gamma_L26_3R_gamma_12C_3R_gamma_19F_3R_gamma_P78048_3R_gamma_P37037_3R_gamma_P37039_3R_gamma_P57055_3R_gamma_P76067_3R_gamma_P76067_4L_gamma
P75063_2L_alpha
P57055_1R_alpha
P87_4R_alpha_P75016_4R_alpha_P75063_4R_alpha
529L_1R_alpha
P57055_2L_alpha
P60002_2L_alpha
GC75_3R_gamma
P94015_2L_alpha_GC75_2L_alpha_P75010_2L_alpha
P37005_1R_alpha
P37039_7R_gamma
SC5314_4L_TLOa9_alpha
P37039_4R_alpha
L26_1R_alpha
WO.1_1L_gamma
P75010_1R_alpha
P34048_1R_alpha
SC5314_7R_TLOg16_gamma_P37005_7R_gamma_L26_7R_gamma_12C_7R_gamma_P60002_7R_gamma_19F_7R_gamma_P78048_7R_gamma_P37037_7R_gamma
P78042_2L_alpha
SC5314_1R_TLOg4_gamma
P78042_4R_gamma
WO.1_1R_alpha
12C_6L_gamma
P76055_6L_truncated
SC5314_6L_TLOg13_gamma_WO.1_RL_gamma_P37005_2L_gamma_P37005_6L_gamma_P87_1L_gamma_19F_6L_gamma_GC75_6L_gamma_P37039_2L_gamma_P37039_6L_gamma_P75016_6L_gamma_P75063_6L_gamma
P75010_6L_gamma
SC5314_2L_TLOg5_gamma_12C_2L_gamma_19F_2L_gamma_P78048_2L_gamma_P76067_2L_gamma
P37005_RL_gamma
P94015_4L_alpha
P78048_6L_gamma
P37037_6L_gamma
P60002_1L_alpha
P78048_3L_gamma
P75016_1L_alpha
GC75_4R_truncated
P75063_3R_gamma
529L_5R_gamma
SC5314_3L_TLOg7_gamma_P37005_3L_gamma_12C_3L_gamma_19F_3L_gamma_GC75_3L_gamma_P34048_3L_gamma_P37039_3L_gamma_P76055_3L_gamma_P76067_3L_gamma
P78042_1R_alpha
P60002_4R_truncated
P76055_4R_truncated
P94015_1R_alpha
GC75_1R_alpha_P57072_1R_alpha
P75010_5L_gamma
P60002_RL_truncated
P78042_RL_alpha
P75016_RL_alpha
P76055_RL_alpha
SC5314_RL_TLOa1_alpha_12C_RL_alpha_GC75_RL_alpha_P78048_RL_alpha_P37037_RL_alpha_P37039_RL_alpha
P76055_2L_alpha
529L_1L_truncated
L26_1L_alpha
P34048_2L_alpha
P76067_RL_alpha
SC5314_1L_TLOa3_alpha_P37005_1L_alpha_12C_1L_alpha_19F_1L_alpha_P78048_1L_alpha_P37037_1L_alpha_P37039_1L_alpha_P75010_RL_alpha_P75010_1L_alpha
P75016_5R_truncated
SC5314_RR_TLOb2_beta_12C_RR_beta_P60002_RR_beta_P78048_RR_beta_P37039_RR_beta_P75016_RR_beta_P57055_RR_beta
P37005_RR_beta
L26_RR_beta
P75063_RR_beta
P57072_RR_beta_P78042_RR_beta
P75010_RR_beta_529L_RR_beta
GC75_RR_beta
P87_RR_beta
P76055_RR_beta
P34048_RR_beta
19F_RR_beta
P94015_RR_beta
WO.1_RR_beta
P37037_RR_beta
0.03
