# Supplementary material for: Architectural groups of a subtelomeric gene family evolve along distinct paths in Candida albicans
Source: G3 (Bethesda). 2022 Oct 21;12(12):jkac283. doi: 10.1093/g3journal/jkac283 (PMC9713401; doi:10.1093/g3journal/jkac283)
Supplement: jkac283_Supplementary_Table_S1 [file jkac283_supplementary_table_s1.pdf]

Supplemental Table 1. TLO sequence architectures are not defined by their MLST genotype group.

Patient isolate	Number of *TLO* α-like sequences	Number of *TLO* β-like sequences	Number of TLOγ-like sequences	Truncated sequences	MLST clade
GC75	6	1	4	**1**	**SA**
P75016	5	1	1	**2**	**SA**
P75063	3	1	2	0	**SA**
P87	2	1	2	0	**SA**
P60002	3	1	1	**2**	**SA**
P94015	3	1	0	**1**	**I**
P34048	5	1	1	0	**III**
P78042	5	1	2	0	**III**
P57055	4	1	1	0	**III**
P57072	3	1	0	0	**II**
P76055	4	1	1	**2**	**II**
P76067	3	0	4	0	**II**
P75010	5	1	2	0	**E**
19F	3	1	6	0	**I**
L26	4	1	3	0	**I**
P37039	5	1	6	0	**I**
12C	5	1	6	0	**I**
P37005	5	1	5	0	**I**
P37037	3	1	4	**1**	**I**
P78048	4	1	6	0	**I**
SC5314	6	1	7	0	**I**
529L	1	1	2	**1**	**N/A**
WO.1	2	1	2	0	**N/A**
