# Supplementary material for: Architectural groups of a subtelomeric gene family evolve along distinct paths in Candida albicans
Source: G3 (Bethesda). 2022 Oct 21;12(12):jkac283. doi: 10.1093/g3journal/jkac283 (PMC9713401; doi:10.1093/g3journal/jkac283)
Supplement: jkac283_Supplementary_Table_S2 [file jkac283_supplementary_table_s2.pdf]

Supplemental Table 2. TLOα and TLOβ group architectures contain PLD signatures in the 3' architecture-specific region.

Seq_ID	Amyloid_Core	pWALTZ_Score	Prion_like_Domain
TLOa1	LDMNNILENDESILDGLNMTL	64.3573	NDNTNDEDMLSNMDYEDLKLDDKVPATTDNNLDMNNILENDESILDGLNMTLLDNGDHVN
TLOb2	NGNNTNHVNEEFDVDGFLNQF	70.0589	NLDPDETIDEVPATTDSDLDMNNILENNESILDGLNMTFLDNGNNTNHVNEEFDVDGFLNQFGN
TLOa3	LDMNNILENDESILDGLNMTL	64.3573	NGNTNDEDMLSNMDYEDLKLDDKVPATTDNNLDMNNILENDESILDGLNMTLLDNGDHVN
TLOa9	HATTDNNLDMNNILENDESIL	64.5644	NGNTNGEDMLSNMDYEDLKLDDKVHATTDNNLDMNNILENDESILDGLNMTLLDNGDHVN
TLOa10	HATTDNNLDMNNILENDESIL	64.5644	NDNTNDEDILSNMDYEDLKLDDKVHATTDNNLDMNNILENDESILDGLNMTLLDNGDHVN
1106_RR	NGNNTNHVNEEFDVDGFLNQF	70.0589	NLDPDETIDEVPATTDSDLDMNNILENNESILDGLNMTFLDNGNNTNHVNEEFDVDGFLNQFGN
1106_1R	LDMNNILENDESILDGLNMTL	64.3573	NDNTNDEDILSNMDYEDLKLDDKVPATTDNNLDMNNILENDESILDGLNMTLLDNGDHVN
1106_4R	LDMNNILENDESILDGLNMTL	64.3573	NDNTNDEDMLSNMDYEDLKLDDKVPATTDNNLDMNNILENDESILDGLNMTLLDNGDHVN
18_RR	NGNNTNHVNEEFDVDGFLNQF	70.0589	NLDPDETIDEVPATTDSDLDMNNILENNESILDGLNMTFLDNGNNTNHVNEEFDVDGFLNQFGN
18_1L	LDMNNILENDESILDGLNMTL	64.3573	NGNTNDEDMLSNMDYEDLKLDDKVPATTDNNLDMNNILENDESILDGLNMTLLDNGDHVN
18_1R	LDMNNILENDESILDGLNMTL	64.3573	NDNTNDEDILSNMDYEDLKLDDKVPATTDNNLDMNNILENDESILDGLNMTLLDNGDHVN
18_4L	LDMNNILENDESILDGLNMTL	64.3573	NDNTNVEDMLSNMDYEDLKLDDKVPATTDNNLDMNNILENDESILDGLNMTLLDNGDHVN
18_4R	HATTDNNLDMNNILENDESIL	64.5644	NDNTNDEDILSNMDYEDLKLDDKVHATTDNNLDMNNILENDESILDGLNMTLLDNGDHVN
17_1L	LDMNNILENDESILDGLNMTL	64.3573	NGNTNDEDMLSNMDYEDLKLDDKVPATTDNNLDMNNILENDESILDGLNMTLLDNGDHVN
17_1R	NDEVILDGLNMTLLVNGDHVN	69.6607	NDNTNDEDILSNMEYEDVKLDDKVPATTDNNLDMNNILENDEVILDGLNMTLLVNGDHVN
15_RL	LDMNNILENDESILDGLNMTL	64.3573	NDNTNDEDMLSNMDYEDLKLDDKVPATTDNNLDMNNILENDESILDGLNMTLLDNGDHVN
15_RR	NGNNTNHVNEEFDVDGFLNQF	70.0589	NLDPDETIDEVPATTDSDLDMNNILENNESILDGLNMTFLDNGNNTNHVNEEFDVDGFLNQFGN
15_1L	LDMNNILENDESILDGLNMTL	64.3573	NGNTNDEDMLSNMDYEDLKLDDKVPATTDNNLDMNNILENDESILDGLNMTLLDNGDHVN
15_4L	LDMNNILENDESILDGLNMTL	64.3573	NDNTNDEDILSNMDYEDLKLDDKVPATTDNNLDMNNILENDESILDGLNMTLLDNGDHVN
15_4R	HATTDNNLDMNNILENDESIL	64.5644	NDNTNDEDILSNMDYEDLKLDDKVHATTDNNLDMNNILENDESILDGLNMTLLDNGDHVN
13_RR	NGNNTNHVNEEFDVDGFLNQF	70.0589	NLDPDETIDEVPATTDSDLDMNNILENNESILDGLNMTFLDNGNNTNHVNEEFDVDGFLNQFGN
13_1R	LDMNNILENDESILDGLNMTL	64.3573	NGNTNDEDMLSNMDYEDLKLDDKVPATTDNNLDMNNILENDESILDGLNMTLLDNGDHVN
13_2L	LDMNNILENDESILDGLNMTL	64.3573	NDNTNDEDMLSNMDYEDLKLDDKVPATTDNNLDMNNILENDESILDGLNMTLLDNGDHVN
13_4L	SDMNNILENDESILDGLNMTL	64.007	NDNTNDEDILSNMDYEDLKLDDKVPATTDNNSDMNNILENDESILDGLNMTLLDNGDHVN
19_RR	NGNNTNHVNEEFDVDGFLNQF	70.0589	NLDPDETIDEVPATTDSDLDMNNILENNESILDGLNMTFLDNGNNTNHVNEEFDVDGFLNQFGN
19_4R	LDMNNILENDESILDGLNMTL	64.3573	NSNTNDEDMLSNMDYEDLKLDDKVPATTDNNLDMNNILENDESILDGLNMTLLDNGDHVN
19_7R	LDMNNILENDESILDGLNMTL	64.3573	NDNTNDEDMLSNMDYEDLKLDDKVPATTDNNLDMNNILENDESILDGLNMTLLDNGDHVN
7_RR	NGNNTNHVNEEFDVDGFLNQF	70.0589	NLDPDETIDEVPATTDSDLDMNNILENNESILDGLNMTFLDNGNNTNHVNEEFDVDGFLNQFGN
7_1L	LDMNNILENDESILDGLNMTL	64.3573	NGNTNDEDMLSNMDYEDLKLDDKVPATTDNNLDMNNILENDESILDGLNMTLLDNGDHVN
7_2L	LDMNNILENDESILDGLNMTL	64.3573	NGNTNDEDMLSNMDYEDLKLDDKVPATTDNNLDMNNILENDESILDGLNMTLLDNGDHVN
6_RR	NGNNTNHVNEEFDVDSFLNQF	71.7948	NLDPDETIDEVPATTDSDLDMNNILENNESILDGLNMTFLDNGNNTNHVNEEFDVDSFLNQFGN
6_1L	LDMNNILENDESILDGLNMTL	64.3573	NGNTNDEDMLSNMDYEDLKLDDKVPATTDNNLDMNNILENDESILDGLNMTLLDNGDHVN
12_RL	LDMNNILENDESILDGLNMTL	64.3573	NDNTNDEDMLSNMDYEDLKLDDKVPATTDNNLDMNNILENDESILDGLNMTLLDNGDHVN
12_RR	NGNNTNHVNEEFDVDGFLNQF	70.0589	NLDPDETIDEVPATTDSDLDMNNILENNESILDGLNMTFLDNGNNTNHVNEEFDVDGFLNQFGN
12_1L	LDMNNILENDESILDGLNMTL	64.3573	NGNTNDEDMLSNMDYEDLKLDDKVPATTDNNLDMNNILENDESILDGLNMTLLDNGDHVN
12_1R	LDMNNILENDESILDGLNMTL	64.3573	IGFDINGNTNDEDMLSNMDYEDLQLDDKVPATTDNNLDMNNILENDESILDGLNMTLLDNGDHVNEE
12_2L	LDMNNILENDESILDGLNMTL	64.3573	NDNTNDEDMLSNMDYEDLKLDDKVPATTDNNLDMNNILENDESILDGLNMTLLDNGDHVN
12_5R	LDMNNILENNESILDGLNMTL	67.4629	YEDLKLDDKVPATTDNNLDMNNILENNESILDGLNMTLLDNGDHVNEEFDVDSFLNQFGN
12_7R	LDMNNILENDESILDGLNMTL	64.3573	NDNTNDEDMLSNMDYEDLKLDDKVPATTDNNLDMNNILENDESILDGLNMTLLDNGDHVN
20_RL	LDMNNILENDESILDGLNMTL	64.3573	NDNTNDEDMLSNMDYEDLKLDDKVPATTDNNLDMNNILENDESILDGLNMTLLDNGDHVN
20_RR	NGNNTNHVNEEFDVDGFLNQF	70.0589	NLDPDETIDEVPATTDSDLDMNNILENNESILDGLNMTFLDNGNNTNHVNEEFDVDGFLNQFGN
20_1L	LDMNNILENDESILDGLNMTL	64.3573	NGNTNDEDMLSNMDYEDLKLDDKVPATTDNNLDMNNILENDESILDGLNMTLLDNGDHVN
20_4R	HATTDNNLDMNNILENDESIL	64.5644	NDNTNDEDILSNMDYEDLKLDDKVHATTDNNLDMNNILENDESILDGLNMTLLDNGDHVN
16_RR	NGNNTNHVNEEFDVDGFLNQF	70.0589	NLDPDETIDEVPATTDSDLDMNNILENNESILDGLNMTFLDNGNNTNHVNEEFDVDGFLNQFGN
16_1L	LDMNNILENDESILDGLNMTL	64.3573	NGNTNDEDMLSNMDYEDLKLDDKVPATTDNNLDMNNILENDESILDGLNMTLLDNGDHVN
16_1R	LDMNNILENDESILDGLNMTL	64.3573	IGFDINGNTNDEDMLSNMDYEDLQLDDKVPATTDNNLDMNNILENDESILDGLNMTLLDNGDHVNEE
16_4L	HATTDNNLDMNNILENDESIL	64.5644	NGNTNGEDMLSNMDYEDLKLDDKVHATTDNNLDMNNILENDESILDGLNMTLLDNGDHVN
47_RR	NGNNTNHVNEEFDVDSFLNQF	71.7948	NLDPDETIDEVPATTDSDLDMNNILENNESILDGLNMTFLDNGNNTNHVNEEFDVDSFLNQFGN
47_1R	LDMNNILENDESILDGLNMTL	64.3573	NDNTNDEDILSNMDYEDLKLDDKVPATTDNNLDMNNILENDESILDGLNMTLLDNGDHVN
47_2L	LDMNNILENDESILDGLNMTL	64.3573	NDNTNDEDMLSNMDYEDLKLDDKVPATTDNNLDMNNILENDESILDGLNMTLLDNGDHVN
47_7R	LDMNNILENDESILDGLNMTL	64.3573	NDNTNDEDMLSNMDYEDLKLDDKVPATTDNNLDMNNILENDESILDGLNMTLLDNGDHVN
3_RL	LDMNNILENDESILDGLNMTL	64.3573	NDNTNDEDMLSNMDYEDLKLDDKVPATTDNNLDMNNILENDESILDGLNMTLLDNGDHVN
3_RR	NGNNTNHVNEEFDVDGFLNQF	70.0589	NLDPDETIDEVPATTDSDLDMNNILENNESILDGLNMTFLDNGNNTNHVNEEFDVDGFLNQFGN
3_1L	LDMNNILENDESILDGLNMTL	64.3573	NGNTNDEDMLSNMDYEDLKLDDKVPATTDNNLDMNNILENDESILDGLNMTLLDNGDHVN
10_RL	LDMNNILENDESILDGLNMTL	64.3573	NDNTNDEDMLSNMDYEDLKLDDKVPATTDNNLDMNNILENDESILDGLNMTLLDNGDHVN
10_RR	NGNNTNHVNEEFDVDGFLNQF	70.0589	NLDPDETIDEVPATTDSDLDMNNILENNESILDGLNMTFLDNGNNTNHVNEEFDVDGFLNQFGN
10_1L	LDMNNILENDESILDGLNMTL	64.3573	NGNTNDEDMLSNMDYEDLKLDDKVPATTDNNLDMNNILENDESILDGLNMTLLDNGDHVN
10_4R	HATTDNNLDMNNILENDESIL	64.5644	NGNTNGEDMLSNMDYEDLKLDDKVHATTDNNLDMNNILENDESILDGLNMTLLDNGDHVN
11_RL	LDMNNILENDESILDGLNMTL	64.3573	NGNTNDEDMLSNMDYEDLKLDDKVPATTDNNLDMNNILENDESILDGLNMTLLDNGDHVN
11_RR	NGNNTNHVNEEFDVDGFLNQF	70.0589	NLDPDETIDEVPATTDSDLDMNNILENNESILDGLNMTFLDNGNNTNHVNEEFDVDGFLNQFGN
11_1L	LDMNNILENDESILDGLNMTL	64.3573	NGNTNDEDMLSNMDYEDLKLDDKVPATTDNNLDMNNILENDESILDGLNMTLLDNGDHVN
11_1R	LDMNNILENDESILDGLNMTL	64.3573	NDNTNDEDMLSNMDYEDLKLDDKVPATTDNNLDMNNILENDESILDGLNMTLLDNGDHVN
11_2L	LDMNNILENDESILDGLNMTL	64.3573	NDNTNDEDMLSNMDYEDLKLDDKVPATTDNNLDMNNILENDESILDGLNMTLLDNGDHVN
9_RL	LDMNNILENDESILDGLNMTL	64.3573	NDNTNDKDMLSNMDYEDLKLDDKVPATTDNNLDMNNILENDESILDGLNMTLLDNGDHVN
9_RR	NGNNTNHVNEEFDVDGFLNQF	70.0589	NLDPDETIDEVPATTDSDLDMNNILENNESILDGLNMTFLDNGNNTNHVNEEFDVDGFLNQFGN
9_1L	LDMNNILENDESILDGLNMTL	64.3573	NGNTNDEDMLSNMDYEDLKLDDKVPATTDNNLDMNNILENDESILDGLNMTLLDNGDHVN
9_2L	LDMNNILENDESILDGLNMTL	64.3573	NGNTNDEDMLSNMDYEDLKLDDKVPATTDNNLDMNNILENDESILDGLNMTLLDNGDHVN
9_4R	LDMNNILENDESILDGLNMTL	64.3573	NSNTNDEDMLSNMDYEDLKLDDKVPATTDNNLDMNNILENDESILDGLNMTLLDNGDHVN
9_7R	LDMNNILENDESILDGLNMTL	64.3573	NDNTNDEDMLSNMDYEDLKLDDKVPATTDNNLDMNNILENDESILDGLNMTLLDNGDHVN
4_RR	NGNNTNHVNEEFDVDGFLNQF	70.0589	NLDPDETIDEVPATTDSDLDMNNILENNESILDGLNMTFLDNGNNTNHVNEEFDVDGFLNQFGN
4_1R	LDMNNILENDESILDGLNMTL	64.3573	NDNTNDEDILSNMDYEDLKLDDKVPATTDNNLDMNNILENDESILDGLNMTLLDNGDHVN
4_2L	LDMNNILENDESILDGLNMTL	64.3573	NDNTNDEDMLSNMDYEDLKLDDKVPATTDNNLDMNNILENDESILDGLNMTLLDNGDHVN
4_7R	LDMNNILENDESILDGLNMTL	64.3573	NDNTNDEDMLSNMDYEDLKLDDKVPATTDNNLDMNNILENDESILDGLNMTLLDNGDHVN
21_RR	NGNNTNHVNEEFDVDGFLNQF	70.0589	NLDPDETIDEVPATTDSDLDMNNILENNESILDGLNMTFLDNGNNTNHVNEEFDVDGFLNQFGN
21_2L	LDMNNILENDESILDGLNMTL	64.3573	NDNTNDEDMLSNMDYEDLKLDDKVPATTDNNLDMNNILENDESILDGLNMTLLDNGDHVN
21_4R	LDMNNILENDESILDGLNMTL	64.3573	NSNTNDEDMLSNMDYEDLKLDDKVPATTDNNLDMNNILENDESILDGLNMTLLDNGDHVN
21_5L	LDMNNILENDESILDGLNMTL	64.3573	NDNTNDEDMLSNMDYEDLKLDDKVPATTDNNLDMNNILENDESILDGLNMTLLDNGDHVN
14_RL	DMNNILENDELILDGLNMTFL	69.2944	NGNTNDEDMLSNMDYEDLKLDDKVPATTDNNLDMNNILENDELILDGLNMTFLDNGDHVN
14_RR	NGNNTNHVNEEFDVDGFLNQF	70.0589	NLDPDETIDEVPATTDSDLDMNNILENNESILDGLNMTFLDNGNNTNHVNEEFDVDGFLNQFGN
14_1L	LDMNNILENDESILDGLNMTL	64.3573	NGNTNDEDMLSNMDYEDLKLDDKVPATTDNNLDMNNILENDESILDGLNMTLLDNGDHVN
14_2L	LDMNNILENDESILDGLNMTL	64.3573	NDNTNDEDMLSNMDYEDLKLDDKVPATTDNNLDMNNILENDESILDGLNMTLLDNGDHVN
2_RL	LDMNNILENDESILDGLNMTL	64.3573	NGNTNDEDMLSNMDYEDLKLDDKVPATTDNNLDMNNILENDESILDGLNMTLLDNGDHVN
2_1L	LDMNNILENDESILDGLNMTL	64.3573	NGNTNDEDMLSNMDYEDLKLDDKVPATTDNNLDMNNILENDESILDGLNMTLLDNGDHVN
2_7R	LDMNNILENDESILDGLNMTL	64.3573	INGNTDEDMLSNMDYEDLKLDNKVPATTDNNLDMNNILENDESILDGLNMTLLDNGDHVNE
8_RL	LDMNNILENDESILDGLNMTL	64.3573	NDNTNDEDMLSNMDYEDLKLDDKVPATTDNNLDMNNILENDESILDGLNMTLLDNGDHVN
8_RR	NGNNTNHVNEEFDVDGFLNQF	70.0589	NLDPDETIDEVPATTDSDLDMNNILENNESILDGLNMTFLDNGNNTNHVNEEFDVDGFLNQFGN
8_1R	LDMNNILENDESILDGLNMTL	64.3573	IGFDINGNTNDEDMLSNMDYEDLQLDDKVPATTDNNLDMNNILENDESILDGLNMTLLDNGDHVNEE
8_2L	LDMNNILENDESILDGLNMTL	64.3573	NDNTNDEDMLSNMDYEDLKLDDKVPATTDNNLDMNNILENDESILDGLNMTLLDNGDHVN
8_7R	LDMNNILENDESILDGLNMTL	64.3573	NDNTNDEDMLSNMDYEDLKLDDKVPATTDNNLDMNNILENDESILDGLNMTLLDNGDHVN
25_RR	NGNNTNHVNEEFDVDGFLNQF	70.0589	NLDPDETIDEVPATTDSDLDMNNILENNESILDGLNMTFLDNGNNTNHVNEEFDVDGFLNQFGN
25_1R	LDMNNILENDESILDGLNMTL	64.3573	NDNTNDEDILSNMDYEDLKLDDKVPATTDNNLDMNNILENDESILDGLNMTLLDNGDHVN
