# Supplementary material for: Architectural groups of a subtelomeric gene family evolve along distinct paths in Candida albicans
Source: G3 (Bethesda). 2022 Oct 21;12(12):jkac283. doi: 10.1093/g3journal/jkac283 (PMC9713401; doi:10.1093/g3journal/jkac283)
Supplement: jkac283_Supplementary_Table_S3 [file jkac283_supplementary_table_s3.pdf]

## Supplemental Table 3. Assignment of sequences downstream of ORF-disrupting mutations.

| MAY number | Strain name | Arm | seq match | phylogenic position |
|---|---|---|---|---|
| 7 | P60002 | 4R | gamma | gamma |
| 14 | P76055 | 4R | gamma | gamma |
| 12 | GC75 | 4R | gamma | ambiguous |
| 13 | P94015 | 7R | alpha | alpha |
| 9 | P75016 | 1R | alpha | alpha |
| 7 | P60002 | RL | alpha | alpha |
| 25 | 529L | 1L | alpha | alpha |
| 9 | P75016 | 5R | alpha | alpha |
| 3 | P37037 | 4L | alpha | alpha |
| 14 | P76055 | 6L | gamma | gamma |
