# Supplementary material for: Architectural groups of a subtelomeric gene family evolve along distinct paths in Candida albicans
Source: G3 (Bethesda). 2022 Oct 21;12(12):jkac283. doi: 10.1093/g3journal/jkac283 (PMC9713401; doi:10.1093/g3journal/jkac283)
Supplement: jkac283_Supplementary_Table_S4 [file jkac283_supplementary_table_s4.pdf]

Supplemental Table 4. Strains used in this study.

Species	Patient isolate	Strain number	Body site of isolation	MLST fingerprinting clade	Reference
Candida albicans	GC75	MAY12	Oral cavity	SA	Hirakawa et al. 2015.
Candida albicans	P75016	MAY9	Bloodstream	SA	Hirakawa et al. 2015.
Candida albicans	P75063	MAY21	Bloodstream	SA	Hirakawa et al. 2015.
Candida albicans	P87	MAY19	Oral cavity	SA	Hirakawa et al. 2015.
Candida albicans	P60002	MAY7	Bloodstream	SA	Hirakawa et al. 2015.
Candida albicans	P94015	MAY13	Bloodstream	I	Hirakawa et al. 2015.
Candida albicans	P34048	MAY47	Bloodstream	III	Hirakawa et al. 2015.
Candida albicans	P78042	MAY8	Bloodstream	III	Hirakawa et al. 2015.
Candida albicans	P57055	MAY47	Bloodstream	III	Hirakawa et al. 2015.
Candida albicans	P57072	MAY16	Bloodstream	II	Hirakawa et al. 2015.
Candida albicans	P76055	MAY14	Bloodstream	II	Hirakawa et al. 2015.
Candida albicans	P76067	MAY2	Bloodstream	II	Hirakawa et al. 2015.
Candida albicans	P75010	MAY11	Bloodstream	E	Hirakawa et al. 2015.
Candida albicans	19F	MAY6	Vagina	I	Hirakawa et al. 2015.
Candida albicans	L26	MAY17	Vagina	I	Hirakawa et al. 2015.
Candida albicans	P37039	MAY10	Bloodstream	I	Hirakawa et al. 2015.
Candida albicans	12C	MAY15	Oral cavity	I	Hirakawa et al. 2015.
Candida albicans	P37005	MAY18	Oral cavity	I	Hirakawa et al. 2015.
Candida albicans	P37037	MAY3	Oral cavity	I	Hirakawa et al. 2015.
Candida albicans	P78048	MAY8	Bloodstream	I	Hirakawa et al. 2015.
Candida albicans	SC5314	MAY1	Bloodstream	I	Hirakawa et al. 2015.
Candida albicans	529L	MAY25	Oral cavity	N/A	Cuomo et al. 2019.
Candida albicans	WO-1	MAY1106	Bloodstream	N/A	Butler et al. 2009.
