# Supplementary material for: Architectural groups of a subtelomeric gene family evolve along distinct paths in Candida albicans
Source: G3 (Bethesda). 2022 Oct 21;12(12):jkac283. doi: 10.1093/g3journal/jkac283 (PMC9713401; doi:10.1093/g3journal/jkac283)
Supplement: jkac283_Supplementary_Table_S5 [file jkac283_supplementary_table_s5.pdf]

Supplemental Table 5. Oligonucleotides used in this study.

ALO number	Oligo Name	Description	Sequence 5' - 3'
35	pan_*TLO*_at_ATG	Universal primer for amplifying *TLO*s from their start site.	ATGCCAGAAAACCTCCAAAC
36	ChrRL_*TLO*_sequencing	Used to amplify subtelomeric *TLO* sequences. ChrR: 11990->71	AGATGAGAGAAATCAGGGCT
37	ChrRR_*TLO*_sequencing	Used to amplify subtelomeric *TLO* sequences. ChrR: 2282075->94	GGTTCCGCATGTTATGGCAT
38	Chr1L_*TLO*_sequencing	Used to amplify subtelomeric *TLO* sequences. Chr1: 12937->18	TGATGTACAAGTCGCCTGAG
39	Chr2L_*TLO*_sequencing	Used to amplify subtelomeric *TLO* sequences. Chr2: 6802->783	CAATGAAGATGACCTGGTGG
40	Chr2R_*TLO*_sequencing	Used to amplify subtelomeric *TLO* sequences. Chr2: 2222442->61	AGGTATTGGTGGTGTGTCCT
41	Chr3L_*TLO*_Sequencing	Used to amplify subtelomeric *TLO* sequences. Chr3: 16010->15991	TTGGAGACGTTGGCTCTGTT
42	Chr3R_*TLO*_sequencing	Used to amplify subtelomeric *TLO* sequences. Chr3: 1786178->97	GGTGCAAGATCATTGGTCTG
43	Chr4L_*TLO*_sequencing	Used to amplify subtelomeric *TLO* sequences. Chr4: 3365->46	TTCAAGTCGTCCAAGTCGTC
44	Chr4R_*TLO*_sequencing	Used to amplify subtelomeric *TLO* sequences. Chr4: 1496619->01	CATTATTGTGTTGGTGGGG
45	Chr5L_*TLO*_sequencing	Used to amplify subtelomeric *TLO* sequences. Chr5: 3652->33	GCGCCACGACTATACAACAA
46	Chr5R_*TLO*_sequencing	Used to amplify subtelomeric *TLO* sequences Chr5: 1180623->42	GATGCTGGCGATATTGTTGC
47	Chr6L_*TLO*_sequencing	Used to amplify subtelomeric *TLO* sequences. Chr6: 7839->20	TCTGGTTCTGGTTCTGGATC
48	Chr7R_*TLO*_sequencing	Used to amplify subtelomeric *TLO* sequences. Chr7: 941364->83	ATTCCCACAGGAACACCCAA
49	Chr_1L_*TLO*_Sanger	Used to sequence into the *TLO* from chromosome arm 1L	CGCTGGGTATCACAAGAGTG
50	Chr_1R_*TLO*_Sanger	Used to sequence into the *TLO* from chromosome arm 1R	TCACCAAGACTGGGTAGAGC
51	Chr_2L_*TLO*_Sanger	Used to sequence into the *TLO* from chromosome arm 2L	GGGGTATTTGGAATGGTTTG
52	Chr_3L_*TLO*_Sanger	Used to sequence into the *TLO* from chromosome arm 3L	CCTATACATAGCCTTCTGCG
53	Chr_3R_*TLO*_Sanger	Used to sequence into the *TLO* from chromosome arm 3R	CTGTGGTGGCAGGTAATTTT
54	Chr_4L_*TLO*_Sanger	Used to sequence into the *TLO* from chromosome arm 4L	CCCACAAATATTTGTTCACC
55	Chr_5L_*TLO*_Sanger	Used to sequence into the *TLO* from chromosome arm 5L	ATGTCTTCGACGGTATTGCC
56	Chr_5R_*TLO*_Sanger	Used to sequence into the *TLO* from chromosome arm 5R	TGTTCACCTTTGACATGCTC
57	Chr_6L_*TLO*_Sanger	Used to sequence into the *TLO* from chromosome arm 6L	TTTCTGGCCTCCTCTGCCTT
58	Chr_7R_*TLO*_Sanger	Used to sequence into the *TLO* from chromosome arm 7R	CAGGTGTTCGCTGTCTATTC
59	Chr_RR_*TLO*_Sanger	Used to sequence into the *TLO* from chromosome arm RR	AAAGCATCTGTAGACACGGC
60	Chr1R_*TLO*_sequencing	Used to amplify subtelomeric *TLO* sequences. Chr1:3184766->86	GGGTTTTTGTTAGTAGCCCAG
61	Chr_RL_*TLO*_Sanger	Used to sequence into the TLO from chromosome arm RL	GGAGTACAGAAGTAGAGCAA
