# Supplementary material for: Architectural groups of a subtelomeric gene family evolve along distinct paths in Candida albicans
Source: G3 (Bethesda). 2022 Oct 21;12(12):jkac283. doi: 10.1093/g3journal/jkac283 (PMC9713401; doi:10.1093/g3journal/jkac283)
Supplement: jkac283_Supplementary_Table_S6 [file jkac283_supplementary_table_s6.pdf]

Supplemental Table 6. Constraint analysis by architecture and chromosomal position.

Group architecture	Alignment			
**Constraint**	full dataset p	LogL	full dataset minus truncated	LogL minus truncated
**none (best)**	0.693	-2983.985113	0.714	-2905.767386
alpha mono	**0.0152**	-3054.839025	**0.0252**	-2948.926326
beta mono	0.583	-2986.888369	0.544	-2910.756334
gamma mono	0.134	-3020.200928	**0.00209**	-2950.459315
beta gamma both mono	0.134	-3020.20091	0.126	-2943.273528
truncated mono	**0.0208**	-3030.799357	N/A	N/A
truncated free other arch. mono	0.0861	-3024.148837	N/A	N/A
group architectures each mono	**0.00358**	-3069.540225	0.0504	-2947.726281

p-AU reported: p-value of approximately unbiased (AU) test (Shimodaira, 2002)

Loci	Alignment	
**Constraint**	full dataset p	LogL
**none (best)**	0.746	-2984.133561
RL mono	**0.0183**	-3051.304722
RR mono	0.542	-2989.870707
1L mono	**0.00488**	-3047.029957
1R mono	**0.0282**	-3045.237273
2L mono	**0.0429**	-3017.641798
3L mono	0.476	-2992.669852
3R mono	0.11	-3001.045603
4L mono	**4.35E-09**	-3081.035712
4R mono	**0.000152**	-3051.990453
5L mono	0.0644	-3033.805039
5R mono	**0.000112**	-3058.432465
6L mono	0.316	-3002.590649
7R mono	**0.0304**	-3030.495945
